# Monocyte to High-Density Lipoprotein Ratio Is Associated With Early Neurological Deterioration in Acute Isolated Pontine Infarction

**DOI:** 10.3389/fneur.2021.678884

**Published:** 2021-06-28

**Authors:** Xinwei Bi, Xiaoqian Liu, Jiaqi Cheng

**Affiliations:** ^1^Department of Neurology, Beijing Shijitan Hospital, Capital Medical University, Beijing, China; ^2^Department of Pharmacy, Affiliated Hospital of Shandong University of Traditional Chinese Medicine, Jinan, China

**Keywords:** acute isolated pontine infarction, early neurological deterioration, monocyte to high-density lipoprotein ratio, monocyte, high-density lipoprotein

## Abstract

**Objectives:** The monocyte to high-density lipoprotein ratio (MHR) has been considered to be a novel inflammatory marker of atherosclerotic cardiovascular disease. However, its role in the acute phase of acute isolated pontine infarctions remains elusive. We explored whether an association existed between elevated MHR levels and early neurological deterioration (END) in patients with isolated pontine infarction.

**Methods:** Data from 212 patients with acute isolated pontine infarction were retrospectively analyzed. We examined the MHR in quartiles of increasing levels to evaluate for possible threshold effects. END was defined as an elevation in the total National Institutes of Health Stroke Scale (NIHSS) score ≥2 or an increase in NIHSS score ≥1 in motor power within the first week after symptom onset. Patients were divided into an END group and a non-END group. The association of MHR on END following pontine infarction was examined by logistic regression models after adjusting for age, NIHSS at admission, basilar artery stenosis, history of hypertension or hyperlipidemia or stroke, infarct size, fasting blood glucose, and paramedian pontine infarction.

**Results:** The mean MHR was 0.44 ± 0.22. A total of 58 (27.36%) patients were diagnosed with END. END occurred within the first 48 h after hospitalization in 38 patients (65.52%). After adjusting for confounding and risk factors, the multivariate logistic regression analysis showed NIHSS at admission [odds ratio (OR), 1.228; 95% confidence interval (CI), 1.036–1.456], basilar artery stenosis (OR, 2.843; 95% CI, 1.205–6.727), and fasting blood glucose (OR, 1.296; 95% CI, 1.004–1.672) were independently associated with END. The odds ratio of END increased as the quartile level of MHR increased, with the lowest quartile used as the reference value. Compared to the first quartile of MHR, the third and fourth quartiles were associated with 4.847-fold (95% CI, 1.532–15.336) and 5.824-fold (95% CI, 1.845–18.385) higher odds of END in multivariate analysis.

**Conclusions:** Elevated MHR levels may be valuable as a biomarker of END in patients with isolated pontine infarction. The elevated MHR was independently associated with END in isolated pontine infarction.

## Introduction

Worsening neurological deficits, also known as early neurological deterioration (END), occur in up to one-third of patients with acute ischemic stroke and have been shown to be associated with increased mortality and subsequent functional disabilities (1, 2). Pontine infarctions account for ~7% of all ischemic strokes, and isolated pontine infarctions are the most common type related to the posterior circulation, accounting for ~15% of cases (3). Extensive studies regarding END prediction in isolated pontine infarction have been performed to enable physicians to better predict END occurrence (4–6). With the popularity of magnetic resonance imaging (MRI) in clinical practice, the correlation between neurological impairment and topographic location has been deeply studied (7, 8). There are also some studies concerning the treatment and prognosis of ischemic stroke (9, 10). However, there are few studies on hematological indexes in the study of the aggravation of nervous system function. Recently, the monocyte to high-density lipoprotein ratio (MHR) has been considered to be a novel inflammatory marker of atherosclerotic cardiovascular disease, especially coronary artery disease (11). It has been reported to be related to the prediction of ischemic stroke from the general population (12) and carotid artery intima-media thickness in patients with type 2 diabetes (13). In stroke-related studies, MHR has been reported to be a good predictive value of stroke-associated pneumonia (14) and mortality in patients with ischemic stroke (15). However, there are no studies exploring the value of MHR in predicting END in patients with acute isolated pontine infarction. Therefore, the aim of our study is to elucidate the association between MHR with END after acute isolated pontine infarction.

## Materials and Methods

### Patients

**Figure 1:**
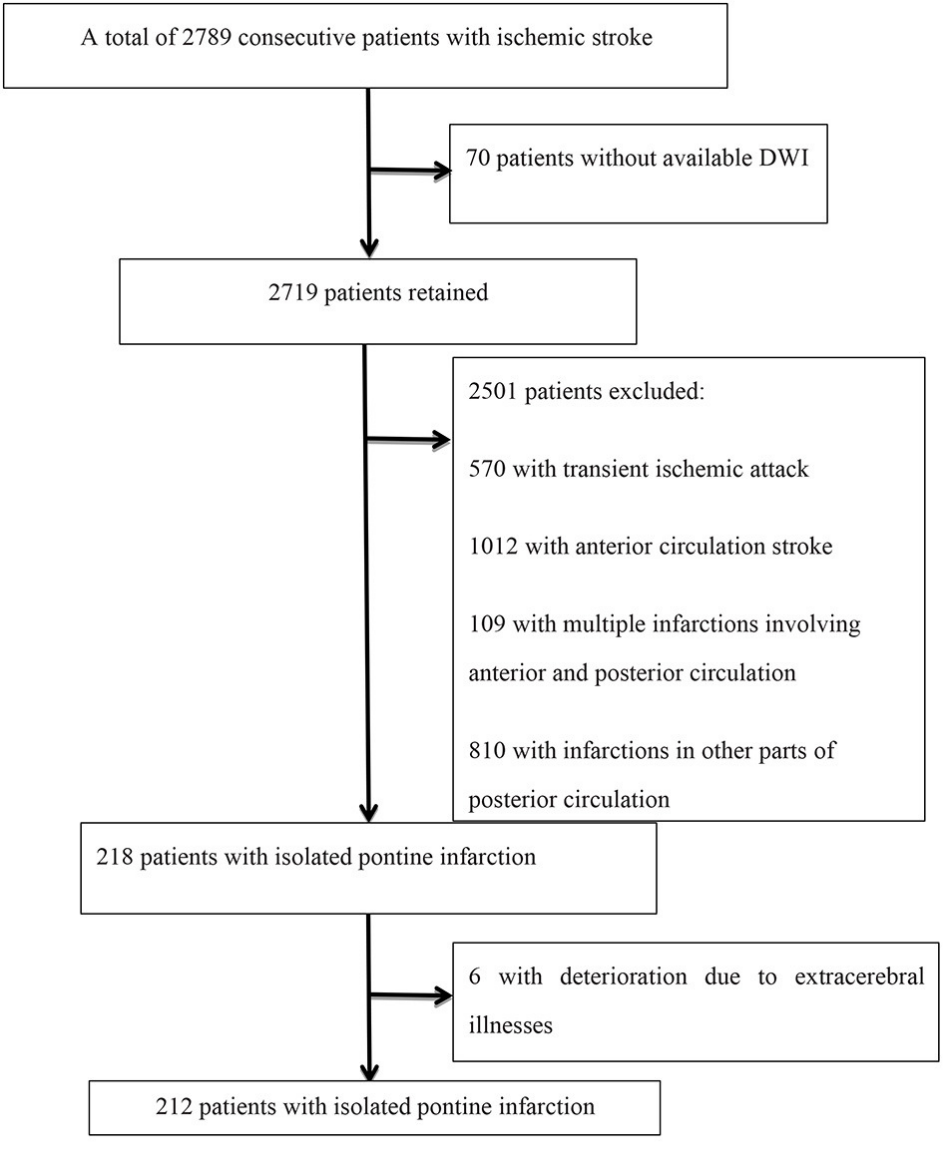
Flowchart of study population inclusion and exclusion.

A total of 2,789 consecutive patients with ischemic stroke registered at the Department of Neurology, Beijing Shijitan Hospital, Capital Medical University from January 2015 to December 2020 were retrieved. Patients were included in the analysis if they met the following criteria: (a) hospitalization within 48 h after the onset of symptoms, (b) acute ischemic lesions within the unilateral pons on diffusion-weighted imaging (DWI), and (c) modified Rankin scale (mRS) score <2 before admission. Patients were excluded if they (a) had no available DWI within 72 h of initial presentation, (b) had infraction of the anterior circulation infarction and/or other parts of the vertebrobasilar system on DWI, and (c) had deterioration due to extracerebral illnesses, such as infection, aspiration pneumonia, hypotension, metabolic disturbances, dehydration, and/or respiratory/heart failure. A flow chart of patient inclusion is shown in Figure 1.

### Clinical Information and Assessment

The following clinical data were retrospectively obtained: age, sex, and vascular risk factors including diabetes mellitus, hypertension, coronary heart disease, hyperlipidemia, previous stroke, and the presence of current smoking. On admission, all patients received brain MRI, magnetic resonance angiography (MRA), carotid artery color Doppler ultrasound, and transcranial Doppler. The following criteria were considered to be vascular risk factors: history of stroke was defined as prior ischemic stroke or transient ischemic attack. Smoking was defined as smoking ≥1 cigarette per day continuously for at least 1 year.

Blood samples were collected on the second day in the morning within 24 h of hospital admission after an 8-h fasting period. Biochemical variables, including serum high-density lipoprotein cholesterol (HDL-C), were measured using an AU5832 automatic biochemical analyzer (Beckman Coulter, Tokyo, Japan). White blood cell (WBC) and monocyte levels were analyzed using a Xe5000 automatic hematology analyzer (SYSMEX, Kobe, Japan). The MHR was calculated as the ratio of the monocyte (× 10^9^/L) count to HDL-C (mmol/L) level.

Severity of neurological impairment was assessed using the National Institutes of Health Stroke Scale (NIHSS) score immediately before MRI scans on admission, within the first 7 days after symptom onset. END was defined as an elevation in the total NIHSS score ≥2 or an increase in NIHSS score ≥1 in motor power within the first week after symptom onset (7). Patients were divided into an END group and a non-END group based on the incremental increase in NIHSS score. The neurological status of patients was evaluated by trained neurologists on a daily basis.

#### Imaging Protocol and Morphometric Analysis

**Figure 2:**
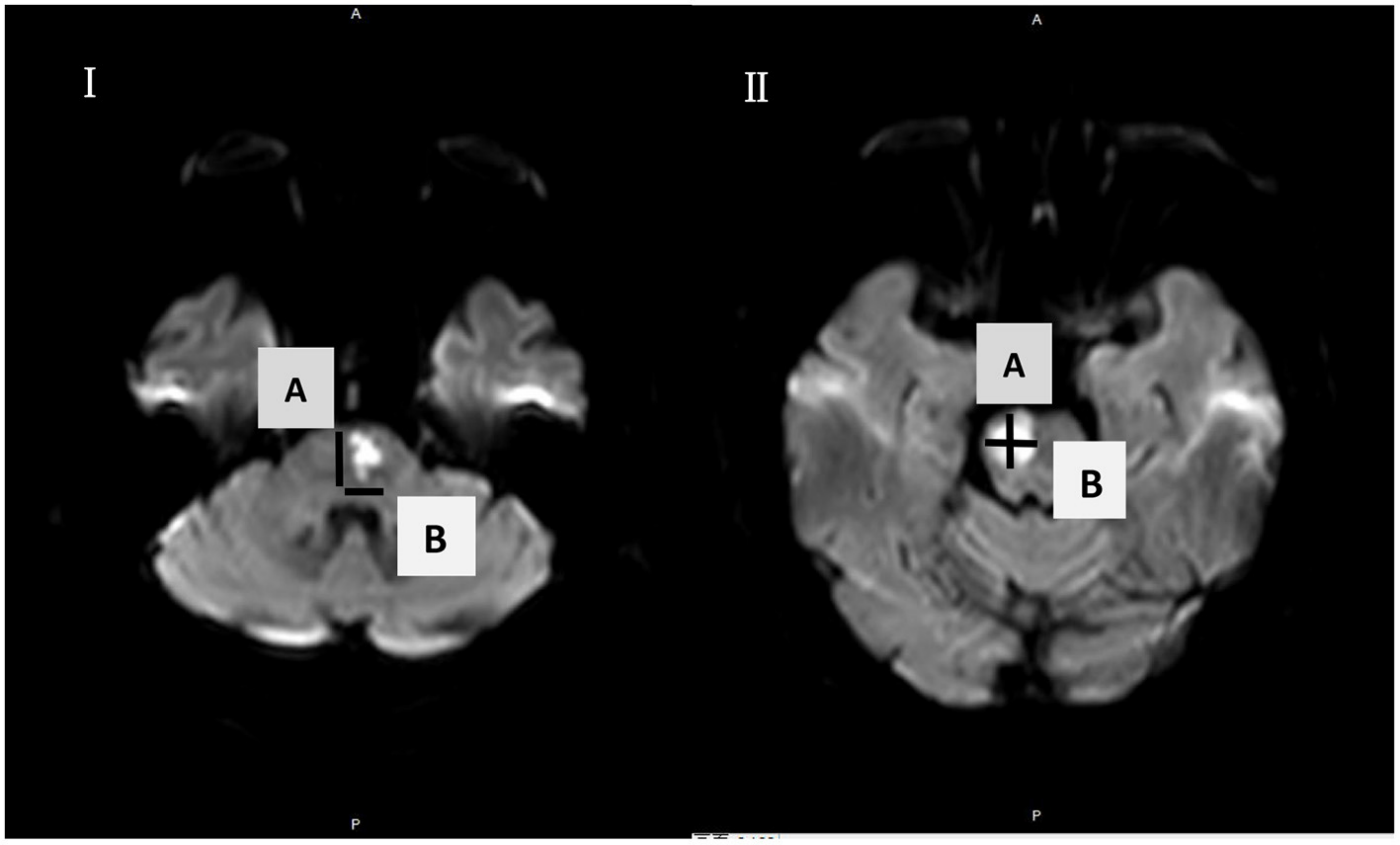
Acute pontine infarctions on axial diffusion-weighted imaging are shown in I and II. The maximal ventrodorsal **(A)** length and **(B)** width of each infarct were measured on axial MRI. A × B was used to define the infarction size (IS).

MRI was performed within 48 h after admission using a 3.0-T MRI unit (Ingenia, Philips, Best, the Netherlands). Morphometric measurement was performed on axial DWI with the following imaging parameters: repetition time = 2,800 ms, echo time = 90 ms, slice/gap = 5 mm/0.5 mm, voxel = 1.0 × 1.0 mm^2^, field-of-view = 230 × 230 mm^2^, and scan time = 35 min. The diffusion sensitivity coefficient B was set to 0 and 1,000 s/mm^2^. DWI was positive for DWI with a high b value and had low a signal according to the apparent diffusion coefficient (ADC). Infarct size (IS) was measured at the axial position on DWI. The maximal ventrodorsal length (A) and width (maximum dimension in a direction perpendicular to the ventrodorsal length, B) of each infarct on axial DWI were measured. We used A × B to represent IS. All morphometric measurements were performed twice, and the mean value was used (Figure 2).

Basilar artery stenosis was defined as a reduction in the caliber of the basilar artery by at least 50% or occlusion of the basilar artery. Isolated pontine infarctions were divided into paramedian pontine infarction (PPI) and lacunar pontine infarction (LPI) (16). PPI was defined as a lesion that extends to the anterior surface of the pons, and LPI was defined as a lesion that does not extend to the basal surface of the pons. The morphometric analysis was performed by at least two neurologists and radiologists.

### Statistical Analysis

We examined the total MHR in quartiles of increasing levels to evaluate for possible threshold effects. Patients were divided into quartiles based on the MHR (Q1, <0.24; Q2, 0.24–0.42; Q3, 0.43–0.55; and Q4, ≥0.56). For group comparisons, analysis of variance or the Kruskal–Wallis rank-sum test was used to compare continuous variables, and the chi-square test was applied for categorical variables. According to the END and non-END groups, baseline characteristics and risk factors were compared using Student's *t*-test (continuous variables) or the χ^2^ test or Fisher's exact test (categorical variables), as appropriate. Continuous variables and categorical variables are expressed as mean (± SD) and frequency (percentage), respectively. Multivariate analyses were performed to determine independent factors associated with END, and the lowest quartile was used as the reference. Considering the close correlation between MHR and monocyte count and HDL level, only the MHR was included in the logistic regression. A receiver-operating characteristic curve was constructed to assess the sensitivity, specificity, and area under the curve of possible contributing factors to discriminate the END group from the non-END group. Spearman correlation was used to judge the relationship between the level of MHR and NIHSS. SPSS version 23.0 for Windows was used for statistical analysis. A two-sided *P* < 0.05 was considered statistically significant.

## Results

Among 2,789 consecutive patients with ischemic stroke, 218 (7.82%) were diagnosed with an isolated pontine infarction, 6 of whom experienced aggravation due to extracerebral illness. A total of 212 patients with acute isolated pontine infarctions were included in the final analysis. The mean age of patients was 68.27 ± 11.57 years, and 127 (59.9%) were male. The mean NIHSS score was 3.59 ± 2.25. A total of 41 (19.3%) patients had basilar artery stenosis. END was diagnosed in 58 (27.36%) patients. END occurred within the first 48 h after admission in 38 patients (65.52%). The mean MHR level was 0.44 ± 0.22. Detailed demographic data are summarized in Table 1. As expected, the presence of basilar artery stenosis (*P* = 0.028), hypertension (*P* = 0.028), and END (*P* = 0.001) was significantly different between groups.

**Table 1:** Characteristics of patients with isolated pontine infarction according to monocyte to high-density lipoprotein ratio quartile.

**Characteristic**	**MHR**				***P***
	**<0.24 (*N* = 52)**	**0.24–0.42 (*N* = 55)**	**0.43–0.55 (*N* = 53)**	**≥0.56 (*N* = 52)**	
Age, years	67.89 ± 11.54	67.33 ± 13.74	71.28 ± 10.96	66.50 ± 9.19	0.157
Sex, male	39 (31.2)	30 (32.9)	31 (31.8)	27 (31.2)	0.071
**Risk factors**					
Current smoking	13 (25.0)	16 (29.1)	16 (30.2)	13 (52.0)	0.899
History of hypertension	28 (53.8)	18 (32.7)	32 (60.4)	25 (25.3)	0.028
History of diabetes mellitus	14 (26.9)	19 (34.5)	24 (45.3)	15 (28.8)	0.188
History of stroke	5 (9.6)	9 (16.4)	9 (17.0)	11 (21.2)	0.461
History of atrial fibrillation	3 (5.8)	1 (1.8)	5 (9.4)	5 (9.6)	0.277
History of coronary heart disease	16 (30.8)	12 (21.8)	12 (22.6)	7 (13.5)	0.21
History of hyperlipidemia	37 (33.4)	33 (35.3)	33 (34.0)	33 (33.4)	0.656
Basilar artery stenosis	4 (7.7)	10 (18.2)	11 (20.8)	16 (30.8)	0.028
Initial SBP, mmHg	147.69 ± 16.58	144.58 ± 18.30	140.87 ± 16.70	141.56 ± 16.43	0.155
Initial DBP, mmHg	83.25 ± 8.78	82.85 ± 8.84	80.96 ± 8.22	82.23 ± 9.19	0.559
NIHSS at admission	3.44 ± 2.08	3.18 ± 2.03	3.71 ± 2.35	4.02 ± 2.51	0.254
HbA_1C_	5.64 ± 0.95	5.77 ± 0.88	5.57 ± 0.89	5.95 ± 1.18	0.243
IS, mm^2^	1.16 ± 0.56	0.88 ± 0.57	0.94 ± 0.58	1.32 ± 0.89	0.11
FBG, mmol/L	6.05 ± 1.21	5.95 ± 1.08	6.09 ± 1.43	6.40 ± 1.83	0.914
TOB, h	18.71 ± 11.84	20.29 ± 11.37	21.94 ± 11.19	22.40 ± 12.10	0.352
END	6 (11.5)	11 (20.0)	18 (34.0)	23 (44.2)	0.001

*MHR, monocyte to high-density lipoprotein ratio; END, early neurological deterioration; SBP, systolic blood pressure; DBP, diastolic blood pressure; PPI, paramedian pontine infarction; LPI, lacunar pontine infarction; NIHSS, National Institutes of Health Stroke Scale; HbA1C, glycosylated hemoglobin; TOB, time from onset to blood sample collection*.

Patient baseline clinical characteristics in the END and non-END groups are shown in Table 2. Basilar artery stenosis (*P* = 0.002), NIHSS at admission (*P* < 0.001), previous stroke (*P* = 0.049), and PPI (*P* = 0.001) were significantly higher in the END group than in the non-END group. Prevalence of hypertension, diabetes, coronary heart disease, hyperlipidemia, atrial fibrillation, and current smoking showed no significant difference between the two groups.

**Table 2:** Comparison of demographic and clinical characteristics between the END non-END groups.

**Characteristics**	**All patients (*N* = 212)**	**END (*N* = 58)**	**Non-END (*N* = 154)**	***P***
Age, years	68.27 ± 11.57	68.54 ± 11.80	67.57 ± 11.00	0.587
Sex, male	127 (59.9)	29 (50.0)	98 (63.6)	0.071
BMI, kg/m^2^	23.98 ± 2.01	24.12 ± 2.00	23.61 ± 1.98	0.096
**Risk factors**				
Current smoking	58 (27.4)	20 (34.5)	38 (24.7)	0.153
History of hypertension	103 (48.6)	28 (48.3)	75 (48.7)	0.956
History of diabetes mellitus	72 (34.0)	18 (31.0)	54 (35.1)	0.581
History of stroke	34 (16.0)	14 (24.1)	20 (13.0)	0.049
History of atrial fibrillation	14 (6.6)	6 (10.3)	8 (5.2)	0.215
History of coronary heart disease	47 (22.2)	9 (15.5)	38 (24.7)	0.152
History of hyperlipidemia	136 (64.2)	37 (63.8)	99 (64.3)	0.947
Basilar artery stenosis	41 (19.3)	19 (32.8)	22 (14.3)	0.002
Initial SBP, mmHg	143.67 ± 17.13	144.53 ± 17.79	143.35 ± 16.92	0.655
Initial DBP, mmHg	82.33 ± 8.74	81.53 ± 9.69	82.62 ± 8.37	0.42
NIHSS at admission	3.59 ± 2.25	4.64 ± 2.31	3.19 ± 2.10	<0.001
PPI	91 (42.9)	36 (62.1)	55 (35.7)	0.001
LPI	121 (57.1)	22 (37.9)	99 (64.3)	0.001
TOB, h	20.83 ± 11.63	19.02 ± 10.19	21.52 ± 12.09	0.133

*END, early neurological deterioration; SBP, systolic blood pressure; DBP, diastolic blood pressure; PPI, paramedian pontine infarction; LPI, lacunar pontine infarction; NIHSS, National Institutes of Health Stroke Scale; BMI, body mass index; TOB, time from onset to blood sample collection*.

Patient laboratory test and imaging results are shown in Table 3. Patients in the END group had larger infarct size (*P* = 0.004), higher HR (*P* < 0.001), higher blood glucose level on admission (*P* < 0.001), and higher monocyte count (*P* < 0.001) than those patients in the non-END group. At the same time, compared with that in the non-END group, the HDL level in the END group was lower (*P* = 0.024).

**Table 3:** Laboratory and imaging data in the END and non-END groups.

**Data**	**END (*N* = 58)**	**Non-END (*N* = 154)**	***P***
FBG, mmol/L	6.62 ± 1.73	5.92 ± 1.22	0.006
Platelet, × 10^9^/L	209.15 ± 777.61	227.05 ± 69.90	0.108
D-dimer, ng/dl	168.91 ± 70.45	179.03 ± 74.49	0.372
Cr, μmol/L	71.71 ± 16.86	70.70 ± 15.36	0.680
WBC, × 10^9^/L	6.89 ± 1.85	6.58 ± 1.86	0.282
Neutrophil, × 10^9^/L	4.34 ± 1.55	4.02 ± 1.57	0.176
Monocyte, × 10^9^/L	0.55 ± 0.28	0.41 ± 0.16	0.001
Lymphocyte, × 10^9^/L	1.83 ± 0.79	1.72 ± 0.56	0.335
Hemoglobin, g/L	143.57 ± 36.73	134.61 ± 24.84	0.090
Total protein, g/L	74.93 ± 52.09	71.78 ± 45.45	0.665
HDL, mmol/L	1.01 ± 0.31	1.19 ± 0.57	0.024
LDL, mmol/L	2.29 ± 0.96	2.39 ± 1.02	0.515
MHR	0.57 ± 0.25	0.39 ± 0.19	<0.001
IS, m^2^	1.29 ± 0.78	0.99 ± 0.62	0.004

*FBG, fasting blood glucose; IS, infarct size; BMI, body mass index; WBC, white blood cell; HDL, high-density lipoprotein; LDL, low-density lipoprotein*.

**Figure 3:**
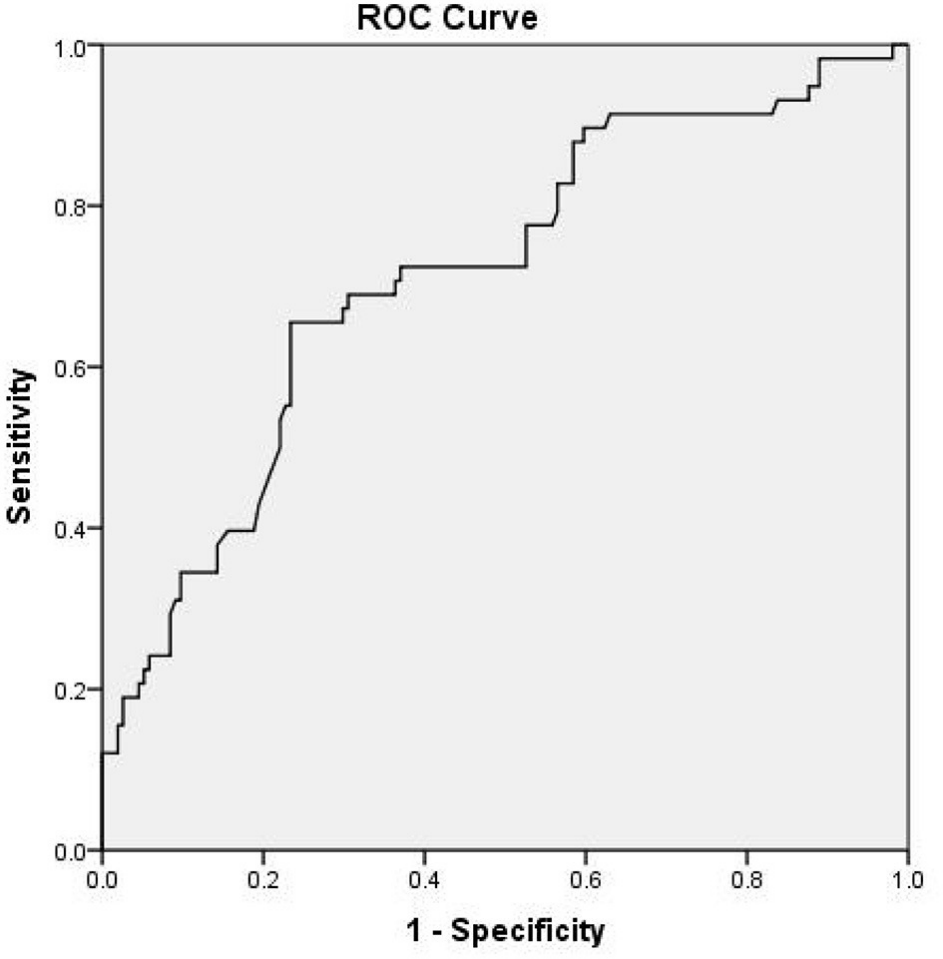
Receiver operating characteristic (ROC) curve of the relationship between monocyte to high-density lipoprotein ratio (MHR) and early neurological deterioration (END).

As presented in Table 4, multivariate logistic regression analysis showed that NIHSS at admission [*P* = 0.018, odds ratio (OR) = 1.228, 95% confidence interval (CI) = 1.036–1.456], basilar artery stenosis (*P* = 0.021, OR = 2.843, 95% CI = 1.205–6.727), and fasting blood glucose (*P* = 0.046, OR = 1.296, 95% CI = 1.004–1.672) were independently associated with END. The odds ratio for END increased with increasing quartile of MHR with the lowest quartile used as the reference value. A high (≥0.43) MHR was independently associated with END (third quartile OR, 4.847; 95% CI, 1.532–15.336; fourth quartile OR, 5.824; 95% CI, 1.845–18.385) in multivariate analysis. The third and fourth highest quartiles of MHR levels were identified to be independently associated with END. Receiver-operating characteristic curve analysis suggested the sensitivity, specificity, and area under the curve for MHR to discriminate END from non-END were 65.5, 76.6, and 71.5% (95% CI, 64–79%; *P* < 0.001), respectively. Youden's index was 0.421. The cutoff was 0.51 (Figure 3).

**Table 4:** Evaluation of the effect of MHR on END following pontine infarction using multivariate logistic regression models.

**Variables**	**OR**	**95% CI**		***P***
NIHSS at admission	1.228	1.036	1.456	0.018
Basilar artery stenosis	2.843	1.205	6.707	0.021
**MHR Q1 (reference)**				
Q2	2.459	0.765	7.9	0.131
Q3	4.847	1.532	15.336	0.007
Q4	5.824	1.845	18.385	0.003
Age	0.987	0.954	1.022	0.466
History of hypertension	0.611	0.274	1.364	0.229
History of hyperlipidemia	1.352	0.583	3.133	0.483
History of stroke	2.463	0.988	6.141	0.053
IS	1.787	0.993	3.215	0.053
FBG	1.296	1.004	1.672	0.046
PPI	1.692	0.758	3.776	0.2

*FBG, fasting blood glucose; IS, infarct size; NIHSS, national institutes of health stroke scale; PPI, paramedian pontine infarction*.

Spearman's correlation was used to determine the relationship between MHR level and NIHSS at 7 days. The results showed that there was a correlation between MHR level and NIHSS at 7 days r_s_ = 0.573, *P* < 0.001.

## Discussion

There are limited reports describing the predictive value of MHR level for early deterioration during the acute phase of an isolated pontine infarction. This study found that elevated MHR levels are associated with END and that the risk of END tended to increase with increasing MHR. It has been unequivocally shown that progressive neurological deficit after ischemic stroke may lead to increased mortality and morbidity (1). Progression of neurologic deficit, however, does not have an authoritative definition, as it could be considered to be either a neuropathological or a clinical event. Early neurological deterioration in patients with isolated pontine infarction is relatively common.

This study showed that 27.36% of the patients with isolated pontine infarction had END, which is consistent with previous studies that reported a prevalence of 20–58% in patients with acute stroke (17, 18). The incidence of END differs between studies according to its definition and the timing and duration of observation (3, 19–21). Although the majority of the relevant studies were based on an increase in NIHSS scores, the exact increase in NIHSS score used to define END has varied drastically. For example, some studies used an NIHSS score increase of 1–2 points combined with motor function impairment to define END, whereas others defined END as an NIHSS increase of 4 points. In terms of the timeframe, END has been defined to occur within 3 days, 5 days, or 1 week after symptom onset in different studies. In our study, END was defined as an elevation in the total NIHSS score ≥2 or an increase in NIHSS score ≥1 in motor power within the first week after symptom onset, which is congruent with most previous studies (7, 22).

Acute inflammation has been observed in brain injury caused by cerebral ischemic diseases, such as the production of inflammatory cells, release of proinflammatory mediators, and tissue infiltration (7, 22). In fact, there is growing evidence that inflammation exerts a prominent effect in the pathogenesis and progression of ischemic stroke (23, 24). A few hours after stroke onset, the number of circulating polymorphonuclear neutrophils increase in a stroke severity-dependent manner (25). Monocytes from the bloodstream reach the damaged site most abundantly 3–7 days after ischemia onset (26). In the early stages after brain injury, the number of total monocytes in the blood circulation shows an increasing trend (27). In addition, previous studies have reported an influx of different immune cells and cytokines produced in the brain, which play an immunomodulatory role in postischemic inflammation (27). Wang et al. suggested that high monocyte counts have the value in predicting the prognosis in various cardiovascular diseases (28). Monocytes play a pivotal role in the initiation and progression of the atherosclerotic process (29). Monocytes in the blood are involved in the start of the process of atherosclerosis by migrating to the intima and differentiating into macrophages under the action of cytokines (30). The increase in the number of macrophages and monocytes around vulnerable plaques can also lead to an increase in the monocyte count in the peripheral blood (31). This inflammatory response takes place during all subtypes of stroke. It could, at least in part, explain the more critical neurological symptomatology and worse outcomes (32). In contrast, HDL-C can control the activation of monocytes while inhibiting the migration of macrophages, protecting endothelial cells from inflammation and oxidative stress (33). Previous studies showed that impaired HDL-mediated cholesterol efflux and low HDL levels caused monocytosis proliferation, leading to a progression of the atherosclerotic plaque (34).

Intracranial atherosclerosis is the main feature of ischemic cerebrovascular disease (35, 36). Branch atherosclerosis, arterial embolism, and hypoperfusion after intracranial atherosclerosis are likely to lead to ischemic stroke (37, 38). Recent studies showed that the occurrence of END was also associated with the severity of basilar artery stenosis (7). By performing autopsies, Caplan identified the basis of pontine infarctions, such as plaque blocking the branch orifice within the parent artery, atherosclerotic plaques originating in the trunk and extending to the branches, and microatheroma originating in the orifice of branches (39). Atherosclerotic stenosis of the basilar trunk was observed in 50% of patients with isolated pontine infarction extending to the basal surface (40). Meanwhile, early neurological deterioration is one of the most concerning clinical problems in patients with branch atherosclerotic diseases. Progressive deficit has been associated with basilar artery branch disease and poor functional outcomes (41). Therefore, the progression of vascular stenosis or thrombosis caused by intracranial atherosclerosis is related to END.

The MHR has recently been used to predict a variety of cardiovascular abnormalities as a developed measure of inflammation and oxidative stress, which reflects the anti-inflammatory and antioxidative effects of HDL, as well as the balance of inflammation and oxidative stress caused by the proinflammatory effects of monocytes. At the same time, MHR has been used as a prognostic indicator in a series of studies. Compared to the control group, patients with acute ischemic stroke had higher MHR, and high values of MHR were found to be a significant independent variable predictive of 30-day mortality in patients with acute ischemic stroke (42). A higher MHR was found to be associated with an increased risk of disability or death at discharge and 3 months after intracerebral hemorrhage, whereas an increase in monocytes was only associated with an increased risk of disability or death after 3 months (43). There are few studies on the association between MHR and acute cerebrovascular disease, especially in the acute phase. We analyzed the correlation between MHR and END in the acute stage of pontine infarction. To our knowledge, this is the first time that inflammatory factors and infarct size have been considered together to study the factors related to END in ischemic cerebrovascular disease. Our study indicated that MHR was an effective and convenient measure in predicting neurological deficit aggravation following pontine infarction. The MHR is a simple and convenient measure that can be effectively applied in clinical practice and provides clinical utility in risk stratification in subjects presenting with isolated pontine stroke. These findings have implications for strategies aimed at lowering the MHR to prevent early neurological progression in patients with ischemic stroke.

Earlier studies have identified the presence of comorbidities (such as diabetes and hypertension), female sex, infarct size, and neurological severity at onset to be associated with progressive deficit in patients with isolated pontine infarctions. However, some studies have reported inconsistent findings (41). In addition, the infarct area extending to the basal surface was 2.5 times greater than deep infarctions without extension to the basal surface (41). Compared to LPI, PPI was related to END in the univariate analysis in our study. In the present study, infarct size had a very high value in the crosstab analysis, which was consistent with many previous studies on infarct size and progression (18); however, in the multiple logistic regression analysis, there was no significant difference in infarct size between the two groups.

In the current study, hyperglycemia correlated with END in patients with isolated pontine infarction after adjusting for other confounding factors in the multivariate analysis. Poststroke hyperglycemia is a common finding among diabetic and nondiabetic patients as a stress response, which is also commonly known as stress hyperglycemia (44). Approximately one-third of stroke patients had hyperglycemia on admission, which was associated with a poor prognosis in patients treated with thrombolytic drugs after ischemic stroke. A recent study reported that stress hyperglycemia increases the risk of severe neurological dysfunction in patients with acute ischemic stroke and is associated with mortality within 1 year (45). Although the exact mechanism underlying the relationship between hyperglycemia and END remains unknown, studies have illuminated the involvement of endothelial injury, tissue acidosis, blood–brain barrier destruction, and production of excessive active oxygen species (46, 47). Therefore, additional studies are necessary to determine whether optimizing blood glucose control could improve the clinical outcomes of patients with pontine infarction.

END following ischemic stroke was a serious event associated with long-term functional outcomes, as reported previously. It was also related to composite event outcomes after discharge during the first year after stroke (48). As inflammatory and immune-mediated mechanisms of neuronal injury have received greater attention, anti-inflammatory treatments have been developed or tested in the preclinical studies and clinical trials, such as IL1-Ra (49, 50), statins (51), and edaravone (52). Despite clinical developments, no beneficial long-term interventions targeting inflammation are currently available.

### Study Limitations

The present study has several limitations. First, this is a single-center retrospective study. Therefore, whether the findings of the present study could be extrapolated to other institutions remains unknown. Moreover, the rate of END in this study is relatively low, further limiting the robustness of the analysis. Our study is confined to isolated brainstem infarction. The next step is to analyze the anterior circulation and posterior circulation, including midbrain and medullary infarctions. Finally, according to previous reports, different inflammatory factors are activated at different times after stroke (24). Some patients may have had the MHR measured at the same time or even after the assessment of END. The change in MHR over time was not studied. Therefore, we only considered a simple association between the biomarker and END instead of any causal relationship. Although statistical associations may exist between some biomarkers and END, the prospective trials are still needed to determine their added value relative to radiological and clinical characteristics.

## Conclusion

In conclusion, patients with acute isolated pontine infarction and elevated MHR levels are at increased risk for END. MHR could be a convenient and effective measure related to END following pontine infarction. In the future, prospective multicenter studies will be needed to conclusively determine the predictive value of MHR for END in acute isolated pontine infarctions.

## Data Availability Statement

The original contributions presented in the study are included in the article/supplementary material, further inquiries can be directed to the corresponding authors.

## Ethics Statement

The studies involving human participants were reviewed and approved by the ethics committees of Beijing Shijitan Hospital, Capital Medical University (No. sjtky11-1x-202091). The participants provided written informed consent to participate in this study. If the participant was unable to provide written informed consent due to illness, the informed consent was signed by the client instead.

## Author Contributions

All authors listed have made a substantial, direct and intellectual contribution to the work, and approved it for publication.

## Conflict of Interest

The authors declare that the research was conducted in the absence of any commercial or financial relationships that could be construed as a potential conflict of interest.
